# Predicting learning and achievement using GABA and glutamate concentrations in human development

**DOI:** 10.1371/journal.pbio.3001325

**Published:** 2021-07-22

**Authors:** George Zacharopoulos, Francesco Sella, Kathrin Cohen Kadosh, Charlotte Hartwright, Uzay Emir, Roi Cohen Kadosh

**Affiliations:** 1 Department of Experimental Psychology, University of Oxford, United Kingdom; 2 Department of Psychology, Swansea University, United Kingdom; 3 Centre for Mathematical Cognition, Loughborough University, United Kingdom; 4 School of Psychology, University of Surrey, Guildford, United Kingdom; 5 School of Psychology, Aston University, United Kingdom; 6 School of Health Sciences, College of Health and Human Sciences, Purdue University, United States of America; UNICOG Cognitive Neuroimaging Lab, FRANCE

## Abstract

Previous research has highlighted the role of glutamate and gamma-aminobutyric acid (GABA) in learning and plasticity. What is currently unknown is how this knowledge translates to real-life complex cognitive abilities that emerge slowly and how the link between these neurotransmitters and human learning and plasticity is shaped by development. While some have suggested a generic role of glutamate and GABA in learning and plasticity, others have hypothesized that their involvement shapes sensitive periods during development. Here we used a cross-sectional longitudinal design with 255 individuals (spanning primary school to university) to show that glutamate and GABA in the intraparietal sulcus explain unique variance both in current and future mathematical achievement (approximately 1.5 years). Furthermore, our findings reveal a dynamic and dissociable role of GABA and glutamate in predicting learning, which is reversed during development, and therefore provide novel implications for models of learning and plasticity during childhood and adulthood.

## Abstract 2

A longitudinal study of human subjects from primary school to university reveals that concentrations of GABA and glutamate in the parietal cortex predict mathematical learning and achievement. During development, the relationships between these neurotransmitters and learning are dynamic, reversing between childhood and young adulthood.

## Introduction

Glutamate and gamma-aminobutyric acid (GABA) have been highlighted as reliable indices of cortical excitability and inhibition, and thus critical for the mechanisms of neuroplasticity and learning [1,2], including development using animal models [3,4]. Moreover, brain excitation and inhibition levels are thought to be critical for triggering the onset of sensitive periods for cognitive skill acquisition by shaping plastic responsiveness of underlying neural systems in response to environmental stimulation [5,6]. Importantly, sensitive periods vary for different functions, with relatively simple abilities (e.g., sensorimotor integration) occurring earlier in development, while the sensitive period for acquiring more complex cognitive functions extends into the third decade of life [5].

Several ^1^H-magnetic resonance spectroscopy (^1^H-MRS, henceforth MRS) studies in human adults have demonstrated the role of glutamate and GABA in plasticity and learning [7–10], in neural activity [11,12], and in sensory and cognitive functions [13–17] using basic lab experiments. However, it is unclear whether and how this knowledge on glutamate and GABA can be applied to more complex human abilities that are slow to emerge. Moreover, it remains to be determined how the link between these neurotransmitters and human learning and cognition varies across development, as suggested by animal models [18–20]. In this respect, educational achievement, such as mathematics (maths), provides a unique cognitive model to examine these questions due to its protracted skill acquisition period that starts already from early childhood and can continue for nearly two decades.

Children receive formal mathematical education from their first year in primary school, and mathematical education can continue into the third decade of age as part of higher education. Mathematical achievement (MA), the performance in mathematical tests, is based on mathematical skills acquisition and is characterized by considerable variability. That is, while some people find maths intuitive and excel in this topic, an estimated 1 in 5 people is considered to have difficulties with maths [21,22]. MA is associated with factors that are central to the welfare of the entire society [23,24], including educational progress [25], socioeconomic status [26], employment, salary, mental and physical health [22], and financial difficulties [27]. As such, MA is the foundation for a thriving society and an important tool for social mobility [26,28].

Previous work suggested that cortical plasticity is underlined by developmental changes in glutamatergic and GABAergic mechanisms, which, in turn, were shown to affect learning and cognitive skills [17,29], making these neurotransmitters excellent candidates for tracking MA across development. To this end, we examined the relationship between GABA and glutamate concentrations in the left intraparietal sulcus (IPS) and the left middle frontal gyrus (MFG), and MA from the beginning of formal schooling to university. Both left and right frontoparietal regions were shown to underpin mathematical processing [30–33]. However, the left hemisphere has shown more consistent involvement in response to mathematical training and education [10,34–39]. We, therefore, focused on the left frontoparietal regions to keep the duration of the study within an acceptable length.

**Fig 1:**
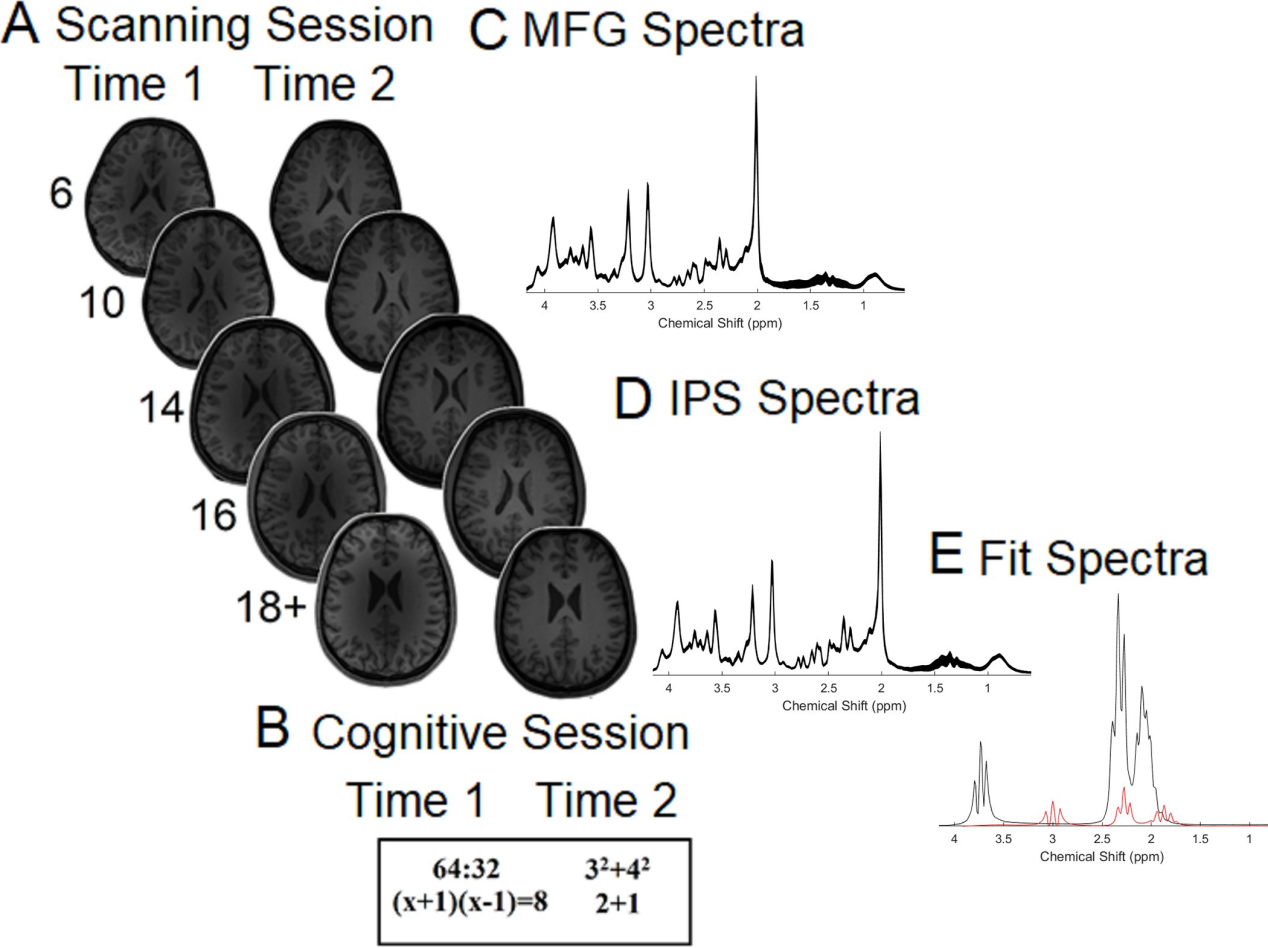
Information about the scanning and cognitive sessions and neurochemical spectra plots. **(A)** Scanning and **(B)** cognitive sessions were completed both during Time 1 and Time 2 (approximately 1.5 years later) in each of the 5 age groups (6-year-olds, 10-year-olds, 14-year-olds, 16-year-olds, and 18+-year-olds). The mean spectra from our sample at Time 1 for the **(C)** MFG and the **(D)** IPS. The thickness corresponds to ±1 SD from the mean (chemical shift expressed in ppm, in x-axis). (**E)** Fit spectra for glutamate (black) and GABA (red) (For the spectra for each of the age groups separately, see **S1 Fig**). The data underlying this figure can be found in **S1 Data**. GABA, gamma-aminobutyric acid; IPS, intraparietal sulcus; MFG, middle frontal gyrus; ppm, parts per million; SD, standard deviation.

Neural plasticity is a rather general term that can take different meanings (i.e., spanning cellular to larger-scale plasticity) and forms, including the formation and elimination of synaptic connections, the modification of synaptic weights, and the reorganization of the brain networks and connections [40,41]. Since we primarily investigate mathematical skills throughout development, here we refer to experience-dependent plasticity, which involves lasting neural changes in response to the environmental input, in this case, formal education [40], and may be underpinned by LTP-like processes, the induction of which primarily involves glutamate and GABA [42]. We focused on the IPS and MFG based on neuroimaging studies demonstrating that mathematical abilities are primarily underpinned by these regions [43–46] and have demonstrated their role in mathematical learning difficulties, exceptional mathematical abilities, and even in basic numerical processing in nonhuman animals [23,34,37,47–52]. We employed a cross-sectional and longitudinal design in 255 participants, ranging from primary school- to university-age. This design allowed us to investigate the link between glutamate and GABA within the IPS and MFG and MA and whether and how it is shaped from early childhood to early adulthood (**Fig 1**). The longitudinal design allowed us to further examine whether neurotransmitter concentration is linked to MA as well as predict MA in the future. Crucially, adopting this design allowed us to discern the selective effect of glutamate and GABA in response to natural (i.e., learning in school) rather than artificial environmental stimulation, thus allowing us to test the knowledge gained from lab-based experiments in high ecological settings. The overall aim of the present study was to examine the capacity of glutamate and GABA within the IPS and MFG in tracking and predicting performance in a complex and slowly emerging cognitive ability, MA, and whether these relations are shaped from early childhood to early adulthood; this aim was achieved.

## Results

### Neurotransmitter measures and MA

#### Age moderated the relation between neurotransmitter concentration and MA

**Fig 2:**
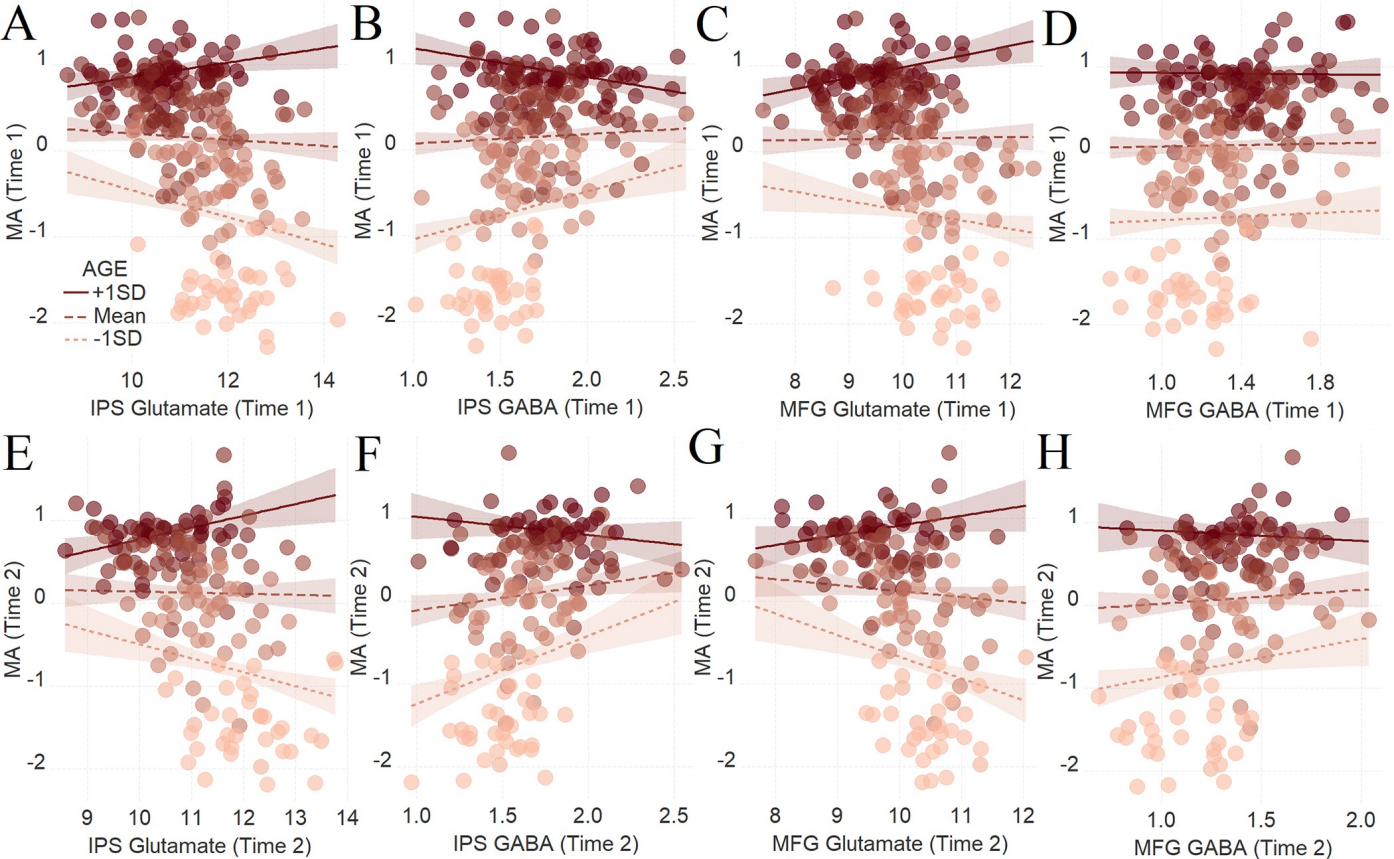
**The moderating role of age in the relation between neurotransmitter concentration and MA at Time 1 (A–D) and at Time 2 (E–H).** To depict the interaction between the continuous variables (age and neurotransmitter concentration), we plotted the regression lines for ± 1 SD from the mean age [53]. Dark color concerns +1 SD above the mean, while light color concerns −1 SD below the mean. The average color represents the mean. (**A)** Glutamate*age and **(B)** GABA*age in the left IPS at Time 1. (C) Glutamate*age and (D) GABA*age in the left MFG at Time 1. (E) Glutamate*age and (F) GABA*age in the left IPS at Time 2. (G) Glutamate*age and (H) GABA*age in the left MFG at Time 2. The shaded area represents 95% confidence intervals. The data underlying this figure can be found in S2 Data. GABA, gamma-aminobutyric acid; IPS, intraparietal sulcus; MA, mathematical achievement; MFG, middle frontal gyrus; SD, standard deviation.

Given the aforementioned aims of our study (i.e., examining learning and achievement across development), we specifically focused on glutamate and GABA, and thus, the other extracted neurochemicals were out of the scope of this study (**S1 Text**). We first examined the association between MA and glutamate and GABA concentrations, and whether such relations are moderated by age, which was indeed the case. In particular, the glutamate concentration in the IPS was negatively associated with MA in younger participants but positively associated with MA in mature participants (**Fig 2A**, β = .13, t(225) = 4.54, standard error (se) = .03, P_HC0_ < .0001, R^2^_ADJ_ = .85, dR^2^_ADJ_ = .01). In contrast, the opposite relationship was found in the same region with GABA, which was positively associated with MA in younger participants but negatively associated with MA in mature participants (**Fig 2B,** β = −.14, t(224) = −5.39, se = .03, P_HC0_ < .0001, R^2^_ADJ_ = .85, dR^2^_ADJ_ = .01). Concerning the MFG, glutamate concentration was negatively associated with MA in younger participants but positively associated with MA in mature participants (**Fig 2C**, β = .11, t(220) = 3.59, se = .03, P_HC0_ = .0004, R^2^_ADJ_ = .85, dR^2^_ADJ_ = .01). Contrary to the IPS, age did not moderate the relationship between GABA concentration and MA in the case of MFG (**Fig 2D**, β = −.02, t(215) = −.58, se = .03, P_HC0_ = .56, R^2^_ADJ_ = .84, dR^2^_ADJ_ = .00).

#### The associations above are not domain-general: MA versus general cognitive ability

We then examined whether our findings were, or were not, domain-general by controlling for general cognitive ability, using matrix reasoning. All the reported results in the case of the IPS remained significant (glutamate*age: β = .09, t(221) = 3.33, se = .03, P_HC0_ = .001; GABA*age: β = −.12, t(220) = −5.21, se = .02, P_HC0_ < .0001). For the MFG, the results were significant for glutamate (glutamate*age: β = .07, t(216) = 2.45, se = .03, P_HC0_ = .015) and not for GABA (GABA*age: β = −.03, t(211) = −.9, se = .03, P_HC0_ = .37). Overall, these results highlighted that the moderating role of age on the relation between IPS GABA and glutamate and MFG glutamate and MA is not confounded by general cognitive ability and thus are not domain-general.

#### Regional and neurotransmitter specificity

To examine the regional as well as neurotransmitter specificity of the IPS glutamate*age and GABA*age in predicting MA, we used a regression model that included all 4 neurotransmitter measures (neurotransmitters (GABA/Glutamate) × region (IPS/MFG)) and their interactions with age, as well as the corresponding main effects. Again, GABA and glutamate interacted with age in the IPS (glutamate*age: β = .11, t(204) = 3.89, se = .03, P_HC0_ = .0001; GABA*age: β = −.14, t(204) = −5.68, se = .02, P_HC0_ < .0001), and glutamate (glutamate*age: β = .08, t(204) = 2.87, se = .03, P_HC0_ = .005), but not GABA (GABA*age: β = .02, t(204) = .75, se = .03, P_HC0_ = .46), interacted with age in the MFG. Therefore, we showed that IPS glutamate and GABA and MFG glutamate had unique contributions when interacting with age in explaining individual variation in MA.

#### Replication of the results at Time 2

We then examined whether the associations we detected at Time 1 are replicated at Time 2. First, we replicated our main finding showing that age moderated the relation between neurotransmitters and MA (**Fig 2E**, IPS glutamate*age: β = .17, t(159) = 4.47, se = .04, P_HC0_ < .0001, R^2^_ADJ_ = .81, dR^2^_ADJ_ = .02; **Fig 2F**, IPS GABA*age: β = −.15, t(159) = −3.84, se = .04, P_HC0_ = .0002, R^2^_ADJ_ = .81, dR^2^_ADJ_ = .02; **Fig 2G**, MFG glutamate*age: β = .16, t(153) = 3.53, se = .05, P_HC0_ = .001, R^2^_ADJ_ = .80, dR^2^_ADJ_ = .02; **Fig 2H**, MFG GABA*age: β = −.08, t(153) = −2.50, se = .03, P_HC0_ = .0135, R^2^_ADJ_ = .79, dR^2^_ADJ_ = .00). Second, we replicated these results even after controlling for the matrix reasoning in the case of IPS (glutamate*age: β = .14, t(158) = 3.64, se = .04, P_HC0_ = .0004; GABA*age: β = −.11, t(158) = −2.64, se = .04, P_HC0_ = .009) and MFG (glutamate*age: β = .12, t(152) = 3.14, se = .04, P_HC0_ = .002; GABA*age: β = −.05, t(152) = −1.41, se = .03, P_HC0_ = .16). Third, we found that our results were regionally and neurotransmitter specific to the IPS. GABA and glutamate in the IPS, but not MFG, explained unique variance of MA (IPS glutamate*age: β = .14, t(143) = 3.16, se = .04, P_HC0_ = .002; IPS GABA*age: β = −.14, t(143) = −3.38, se = .04, P_HC0_ = .001; MFG glutamate*age: β = .08, t(143) = 1.43, se = .05, P_HC0_ = .15; MFG GABA*age: β = −.04, t(143) = −1.21, se = .03, P_HC0_ = .23).

### Neurotransmitter measures and future MA

We then examined whether neurotransmitter concentrations can predict future MA approximately 1.5 years later.

**Fig 3:**
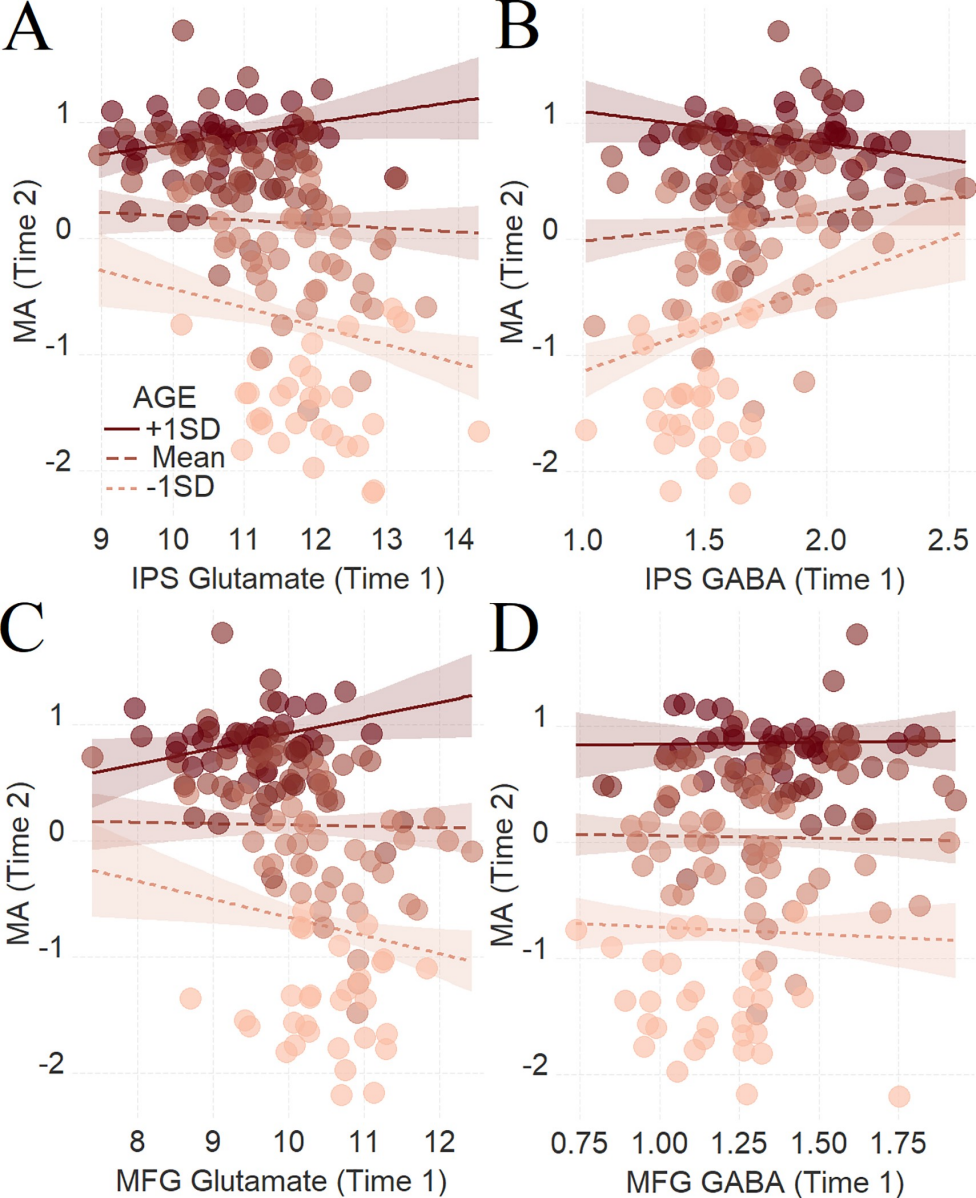
Predicting MA at Time 2 from the interaction between age neurotransmitter concentration at Time 1. To depict the interaction between the continuous variables, we plotted the regression lines for ± 1 SD from the mean age [53]. Dark color concerns +1 SD above the mean, while light color concerns −1 SD below the mean. The average color represents the mean. (**A)** Glutamate and **(B)** GABA in the left IPS; (**C)** glutamate and (**D)** GABA in the left MFG. The shaded area represents 95% confidence intervals. For visualization purposes, the effect of age at Time 2 was not included when generated the panels of this figure. The data underlying this figure can be found in **S3 Data**. GABA, gamma-aminobutyric acid; IPS, intraparietal sulcus; MA, mathematical achievement; MFG, middle frontal gyrus; SD, standard deviation.

We examined whether age at Time 1 moderates neurotransmitters’ effect at Time 1 on future MA as assessed at Time 2. This was the case especially for the IPS (**Fig 3A**, glutamate*age β = .14, t(150) = 3.64, se = .04, P_HC0_ = .0004, R^2^_ADJ_ = .81, dR^2^_ADJ_ = .02; **Fig 3B**, GABA*age: β = −.16, t(149) = −4.45, se = .04, P_HC0_ < .0001, R^2^_ADJ_ = .81, dR^2^_ADJ_ = .02), although we also found the involvement of the MFG glutamate but not the MFG GABA (**Fig 3C**, glutamate*age: β = .14, t(147) = 3.13, se = .04, P_HC0_ = .002, R^2^_ADJ_ = .80, dR^2^_ADJ_ = .01; **Fig 3D**, GABA*age: β = .02, t(143) = .55, se = .04, P_HC0_ = .6, R^2^_ADJ_ = .78, dR^2^_ADJ_ = .00).

Further analysis that included all the neurotransmitters in the regression model indicated that these results are regionally and neurotransmitter specific to the IPS and that GABA and glutamate predicted unique variance in future MA (IPS glutamate*age: β = .14, t(130) = 3.33, se = .04, P_HC0_ = .001; IPS GABA*age: β = −.19, t(130) = −5.43, se = .03, P_HC0_ < .0001; MFG glutamate*age: β = .08, t(130) = 1.94, se = .04, P_HC0_ = .055; MFG GABA*age: β = .05, t(130) = 1.29, se = .04, P_HC0_ = .2).

## Discussion

Our results reveal how glutamate and GABA, the neurotransmitters involved in brain excitation and inhibition [9,54–56], are associated with MA and the feasibility of using them to predict future MA from childhood to adulthood. We provide such understanding by demonstrating regional and neurotransmitter specificity of these neurotransmitters–MA relationships and their moderation by age and showing that these associations are not domain-general. Notably, we also showed that GABA and glutamate levels in the IPS can predict future MA as a function of age.

These results provide a novel insight into the developmentally dependent manner in which educational achievement relates to glutamate and GABA levels, which have been previously associated with markers of cortical excitability and inhibition important for mechanisms of learning and sensitive periods in development [5,9,10,12–15,56]. By using a large sample of participants ranging from primary school age to university students, we were able to show how variations in glutamate and GABA are associated with high-level cognition, namely MA, and that the connection between glutamate and GABA and cognition is altered as a function of development.

The IPS has been highlighted as a key brain region in numerical and mathematical cognition and learning as supported by methodologically diverse studies including nonhuman primates, neurological and neurodevelopmental populations, and mathematical experts based primarily on neurofunctional and structural studies in healthy humans [46,57–61]. Our results expanded this understanding by showing that the developmental stage influences the relation between IPS neurotransmitter concentration and MA. Specifically, mature individuals show a positive association between glutamate concentration and MA, while younger participants show a negative association. This suggests that higher parietal glutamate concentration is related to higher MA later in development, but the opposite is true earlier in development. By including children from the age of 6 to university students and examining this sample approximately 1.5 years later, we showed that the association between MA and IPS glutamate is switched from negative to positive during development.

Furthermore, we were able to extend our findings and show the inverse effect for IPS GABA; mature individuals showed a negative association between GABA concentration and MA, while younger participants show a positive association. Maturation of GABA circuits and in particular that of parvalbumin cells, a positive subtype of GABA neurons, is thought to be one of the molecular signatures triggering the onset of sensitive periods and plasticity whereby experimental increase or reduction of GABA triggers precocious and delayed onset of sensitive periods in animal studies, respectively [5]. Therefore, elevated GABA early in development may indicate a greater plasticity leading to greater MA. Regarding the relation between GABA levels and learning in mature individuals, our findings indicate a negative association. Previous studies on GABA and learning in adults, using modest sample sizes, yielded some conflicting results in that some studies found reduced GABA levels to be associated with learning improvement, while others found the opposite pattern. For example, reduced GABA was associated with learning improvement in the motor system [62–64] and the visual system [14,65], although some types of visual learning, and sensory learning in the tactile system, were associated with increased GABA [13,65–67]. In the realm of cognition, several studies found increased GABA to be indicative of learning improvement [10,68,69], while others found the opposite pattern [70]. However, there are several reasons for these apparent discrepancies. For example, it is important to mention that the target MRS brain regions vary between these studies, and one cortical area may not represent or generalize to other cortical areas concerning the association of neurotransmitter levels and the process under investigation. Besides these discrepancies, our findings in the mature participants suggest that lower GABA concentration within the IPS leads to enhanced learning, thus extending the involvement of GABA in the acquisition of a slowly emerging complex cognitive function in a highly ecological setting.

Taken together, our finding of developmental switches in the link between GABA and glutamate and MA may highlight a general principle of plasticity. According to our findings, GABA and glutamate concentrations enhance or constrain the plasticity of a given cognitive function depending on the sensitive period of that cognitive function (i.e., early sensitive period versus late sensitive period). We suggest that increased GABA levels during the early sensitive period, which based on research in animals [5] may reflect the maturation of paralbumin-based GABAergic networks, lead to greater MA, but that increased GABA levels in the later sensitive period impair MA. Therefore, in contrast to previous studies on humans or animals that focused on narrower developmental stages, our cross-sectional longitudinal study suggests that the link between plasticity and brain excitation and inhibition across different stages is unlikely to be immutable, a finding that has implications for basic and translational research. Crucially, both glutamate and GABA in the IPS, but not in the MFG, explained unique variance in MA during development, a result that demonstrates their dissociated role in the current research context. While MFG glutamate was a significant predictor in some analyses, it was a weaker predictor and less consistent than the IPS GABA and glutamate, which strongly predicted future MA as a function age. Overall, unlike the IPS, the neurochemical contribution of MFG did not track MA to the same extent. Meta-analyses suggested a hierarchical contribution of the prefrontal cortex in numerical cognition [44]. Namely, the inferior frontal gyrus was typically engaged in relatively simple numerical tasks, while the MFG is involved in more complex tasks, which require several procedural steps or increase storage load [44]. This role of the MFG likely reflects shared links to working memory, which is behaviourally related to numerical performance [71,72] and supported by the prefrontal cortex [73,74]. Indeed, a recent functional MRS study found elevated glutamate levels in the dorsolateral prefrontal cortex during the execution of a 2-back task compared to passive visual fixation [75]. Given this contribution of the MFG in demanding computations that mirror the computations underlying the present cognitive tasks at least in case of early childhood, one potential reason we did not find such a strong association between MFG neurotransmitter levels and MA, compared to the IPS, may be accounted for the fact that neurotransmitter levels were measured at baseline rather than during the execution of the numerical tasks. However, our results do not exclude the role of the MFG in mathematical cognition. For example, in a recent study, we showed a reduction in MFG GABA due to the lack of mathematical education in adolescents [10]. However, such finding is orthogonal to the current results that show the link between MA and GABA and glutamate, and the ability of the latter to predict future MA.

In arithmetic problem solving, a frontoparietal network comprising the prefrontal cortex and the IPS (task-positive network) is initially involved due to the reliance on working memory, which is associated with reduced proficiency in children. In contrast, the frontoparietal network becomes less involved with increased proficiency and a shift to fact retrieval strategies involving episodic and semantic memory systems in angular guys and hippocampus, which are critical for long-term memory formation [30,76–78]. This well-documented developmental change in processing strategies underpinned by the shift in the recruitment of large brain circuits may be reflected by the differential relation between glutamate and GABA concentrations in the MFG and IPS, which are key seeds of the frontoparietal networks. One potential mechanism is that the developmental reduction in glutamate and increase in GABA in both the MFG and the IPS from childhood to adulthood may reduce the reliance on the frontoparietal network as the aforementioned semantic and episodic regions become more relevant.

Of note, previous work suggested that glutamate and GABA concentrations might not reflect the same levels of cortical inhibition and excitation across brain development [79]. In particular, it has been shown in the immature nonhuman brain that GABA is excitatory, and GABA-releasing synapses are formed before glutamatergic contacts in a wide range of species and structures [79]. GABA becomes inhibitory by the delayed expression of a chloride exporter [80]. Indeed, from animal research, it is known that in the case of chloride, the reversal potential shifts as the animal matures, in that it is more depolarized in the younger animals (−40 mV) than in adulthood (−65 mV). Critically, MRS cannot currently distinguish between intracellular and extracellular neurotransmitter concentrations or even a portion of these based on the MRS signal alone [81]. Consequently, making direct inferences of cortical excitability/inhibition and plasticity based on the neurotransmitter concentrations alone should be done with caution. Indeed, several potential mechanisms have been proposed in the context of MRS concentration changes for both GABA (e.g., decreased GABA metabolism, increased catabolism, and a shift of GABA into an MRS-invisible pool) and glutamate (e.g., glutamate levels were closely related to transcranial magnetic stimulation measures of local excitability) [42]. Nonetheless, our results suggest that the interaction between IPS glutamate and age and between IPS GABA and age exert differential influences shaping educational achievement, even at the younger age groups.

In addition, our findings allow us to conclude that these associations are not confounded by general cognitive ability. The link between IPS neurotransmitters and MA was still significant when we included matrix reasoning in our multiple regression analysis, concluding that our findings are not domain-general. The value of adding matrix reasoning lies in its link to fluid intelligence, which is associated with mathematical abilities [82]. Our findings may also highlight a general principle that the developmental dynamics of regional excitation and inhibition levels in regulating the sensitive period and plasticity of a given high-level cognitive function (i.e., MA) may be different compared to another high-level cognitive function (i.e., general intelligence) that draws on similar, albeit not identical, cognitive and neural mechanisms.

While our research focused on a healthy population, it motivates further research to understand how alteration in glutamate and GABA are linked to neurodevelopmental deficits [5,83] and whether modulation of those neurotransmitters can improve interventional outcomes [84], potentially by expanding or reopening sensitive period processes [5]. By shedding light on the developmental trajectories in elucidating the effect of glutamate and GABA on educational achievement, and the putative sensitive periods in development where the relationship between these neural and cognitive measures switches, our study provides a novel understanding of the human brain and its impact on formal education.

## Materials and methods

### Participants

We recruited 255 participants (demographic information for both the first assessment and the second assessment is shown in **S1 Table**). The imaging session lasted approximately 60 min, and the mathematical assessment and general cognitive ability testing lasted approximately 30 min; these sessions were part of a more extensive battery that included several other cognitive and behavioral assessments. All imaging data were acquired in a single scanning session. During the scanning acquisition, participants watched the LEGO movie [85]. All participants were predominantly right-handed, as measured by the Edinburgh Handedness Inventory [86] and self-reported no current or past neurological, psychiatric, or learning disability or any other conditions that might affect cognitive or brain functioning. Adult participants received £50 compensation for their time, and children participants, depending on their age, received £25 (6-year-olds) and £35 (10-, 14-, and 16-year-olds) in Amazon or iTunes vouchers, and additional compensation for their caregiver if the participant was below 16 years. Informed written consent was obtained from the primary caregiver, and informed written assent was obtained from participants younger than 16 years, according to approved institutional guidelines. Our sample was reassessed approximately 1.5 years later (mean = 20.97, SD = 3.83 months). We refer to the first assessment as Time 1 and to the second assessment as Time 2. This study was approved by the University of Oxford’s Medical Sciences Interdivisional Research Ethics Committee (MS-IDREC-C2_2015_016) and adhered to the principles expressed in the Declaration of Helsinki. Approximately 31% of the participants who completed the first assessment did not participate in the second assessment.

### MRI data acquisition and preprocessing

All MRI data were acquired using a 3T Siemens MAGNETOM Prisma MRI System equipped with a 32 channel receive-only head coil.

#### Structural MRI

Anatomical high-resolution T1-weighted scans were acquired consisting of 192 slices, repetition time (TR) = 1,900 ms; echo time (TE) = 3.97 ms; voxel size = 1 × 1 × 1 mm).

#### Magnetic resonance spectroscopy

Spectra were measured by semi-adiabatic localization using an adiabatic selective refocusing (semi-LASER) sequence (TE = 32 ms; TR = 3.5 s; 32 averages) [87,88] and variable power RF pulses with optimized relaxation delays (VAPOR), water suppression, and outer volume saturation. Unsuppressed water spectra acquired from the same volume of interest were used to remove residual eddy current effects and to reconstruct the phased array spectra with MRspa (https://www.cmrr.umn.edu/downloads/mrspa/). Two 20 × 20 × 20 mm^3^ voxels of interest were manually centered in the left IPS and the MFG based on the individual’s T1-weighted image while the participant was lying down in the MR scanner (**S2 Fig**). Acquisition time per voxel was 10 to 15 min, including sequence planning and shimming. MRS neurotransmitters were quantified with the LCmodel [89], using a basis set of simulated spectra generated based on previously reported chemical shifts and coupling constants based on a versatile simulation, pulses, and analysis (VeSPA) simulation library [90]. Simulations were performed using the same RF pulses and sequence timings as in the 3T system described above. Absolute neurotransmitter concentrations were extracted from the spectra using a water signal as an internal concentration reference. The exclusion criteria for data were (i) Cramér–Rao bounds and the (ii) signal-to-noise ratio (SNR). Neurotransmitters quantified with Cramér–Rao lower bounds (CRLBs, the estimated error of the neurotransmitter quantification) >50% were classified as not detectable. Additionally, we excluded cases with an SNR beyond 3 standard deviations and a concentration value that was beyond 3 standard deviations at the age-group level per region and neurotransmitter. Absolute neurotransmitter concentrations were then scaled using the structural properties of the selected regions [89]; therefore, the scaling values were determined before the data collection. Namely, we segmented the images into different tissue classes including gray matter (GM), white matter (WM), and cerebrospinal fluid (CSF) using the SPM12 segmentation facility. Next, we calculated the number of GM, WM, and CSF voxels within the 2 masks of interest separately around the left MFG and the left IPS in native space. Subsequently, we divided these 6 numbers (GM, WM, and CSF for IPS and MFG) by the total number of GM, WM, and CSF voxels creating the corresponding GM, WM, and CSF fraction values per participant and region. As a final computation step, we scaled the absolute neurotransmitter values to these structural fractions using the following LCmodel [89] computation as can be seen in MRS-Eq 1:

Tissue−correctedconcentration=((43300/55556*GMfraction+35880/55556*WMfraction+1*CSFfraction)/(1−CSFfraction))*absoluteneurotransmitterconcentration (MRS-Eq 1)

To minimize the potential confounding effects of T2 relaxation times, we additionally report the results when an alternative neurochemical quantification was used (**S2–S5 Tables**). These concentration values were scaled based on the T2 values, as can be seen in MRS-Eq 2. T2 values were acquired by obtaining spectra using 13 different echo times (32 ms, 42 ms, 52 ms, 85 ms, 100 ms, 115 ms, 150 ms, 250 ms, 450 ms, 850 ms, 1,650 ms, 3,250 ms, and 4,040 ms).


$$T2-corrected\;concentration=tissue-corrected\;concentration*exp\;\left(-\frac{TE}{T2}\right)$$ (MRS-Eq 2)

Of note, the mean cross-correlation between the neurotransmitter pairs Glutamate-Gln and Glutamate-GABA in both regions of interest (MFG and IPS) was <.5, suggesting that there was not a significant overlap between these neurotransmitters and allows to report these concentrations separately [91]. For the neurochemicals included in the basis set, please see **S1 Text**. Apart from glutamate and GABA, we did not have any specific predictions about the other extracted neurochemicals in respect to learning and achievement, and, therefore, they were out of the scope of this study.

### MA

Participants completed the numerical operation and the mathematical reasoning subtests of the Wechsler Individual Achievement Test (WIAT), second edition [92], and the tempo test Rekenen [93]. The numerical operation subtest is composed of written arithmetic problems, which require the implementation of arithmetic procedures. The mathematical reasoning subtest is composed of maths problems, which require participants to create a mental model of the math problem, extract relevant information, and then select and execute the appropriate operation [94]. Both tests are completed without a time restriction. We calculated the proportion of correct responses for the numerical operations and the mathematical reasoning subtests. These 2 tests present problems that are ordered with increasing difficulty. Participants start by responding to questions that are appropriate for their age and, in case of 6 consecutive wrong responses, the administration was interrupted. Therefore, young children were unlikely asked to solve questions that were not appropriate for their age.

The tempo test, instead, entails 5 columns (addition, subtraction, multiplication, division, and mixed), each composed of 40 arithmetic problems (e.g., 7 + 8 = ….). Each column is presented sequentially with the instruction to solve as many problems as possible within 60 s. The time constraint makes the tempo test a widely used measure of arithmetic fluency. For the tempo test instead, we calculated the proportion of correct responses in the first 2 columns (additions and subtractions; because only these 2 columns were completed by all age groups), and then we divided this score by the individual solving time divided by total time at disposal (i.e., 300 s). This efficiency score increases in case a participant completed all the arithmetic problems in a column within 60 s. Finally, we z-scored and averaged the above 3 values into a single individual MA score. Such a score allowed us to provide a measure that is not based on a single MA measure. For the results using each of the 3 tests separately, which mainly converge with the main text results, see **S3–S8 Tables**.

### General cognitive ability

Participants completed the matrix reasoning subtest of the Wechsler Abbreviated Scale of Intelligence (WASI II) [95] as an index of fluid intelligence, which has been previously related to MA [96]. Accordingly, MA and the scores in matrix reasoning were highly correlated both at Time 1 (r_S_(248) = .81, *P* < .001) and at Time 2 (r_S_(173) = .8, *P* < .001), and after controlling for age (Time 1: r(247) = .47, *P* < .001; Time 2: r(172) = .43, *P* < .001).

### Statistical analyses

For statistical analyses, we used SPSS (v25), R package (v3.5.3), and MATLAB (R2020a). To assess the moderating role of age, we ran linear regression models, and the effect of interest was the interaction between neurotransmitter concentration and age. To assess the moderating role of age in shaping the relation between neurotransmitter concentration and MA at Time 1, we employed equation 1, and to assess the same association at Time 2, we employed equation 2.


MA(Time1)=β0+β1neurotransmitter(Time1)+β2age(Time1)+β3neurotransmitter(Time1)*age(Time1)+ε

MA(Time2)=β0+β1neurotransmitter(Time2)+β2age(Time2)+β3neurotransmitter(Time2)*age(Time2)+ε

To show that our findings are not domain-general, we reran variants of equation 1 and equation 2 and controlled for the main effect of matrix reasoning score during the respective time of testing by adding it as a covariate. Similarly, to examine the neurotransmitter and neuroanatomical specificity of our findings, we included all 4 neurotransmitter measures (neurotransmitters (GABA/Glutamate) × region (IPS/MFG)) and their interactions with age to equation 1 (for Time 1) and equation 2 (for Time 2). Finally, to examine how age and neurotransmitters at Time 1 predicted MA approximately 1.5 years later, we employed equation 3.


MA(Time2)=β0+β1neurotransmitter(Time1)+β2age(Time1)+β3neurotransmitter(Time1)*age(Time1)+β4age(Time2)+ε

In the main text, we additionally report the adjusted R^2^ (R^2^_ADJ_) for each of the main models, as shown in equations 1, 2, and 3. Since the present study is focused on the confluence of age and neurotransmitter levels, we additionally report the adjusted R^2^ difference (dR^2^_ADJ_) between the models shown in equations 1, 2, and 3 versus the corresponding models when omitting the interaction term.

Since the assumption of homoscedasticity was violated in our analyses (see **S9 Table**), we report *P* values derived from statistical tests that are robust to the assumption of homoskedasticity, which are techniques to obtain unbiased standard errors of ordinary least squares coefficients under heteroscedasticity (HC0 termed P_HC0_) [97,98]. All *P* values in the results section correspond to the interaction term between age and the corresponding neurotransmitter measure. The normality of the residuals assumption was not violated in the main models as assessed with the Shapiro–Wilk test (**S10 Table**). The multicollinearity assumption was also not violated as assessed with the variance inflation factor (**S11 Table**).

When significant interactions were present, we also reported the results using the Johnson–Neyman Technique (JNT), which allowed us to clarify the nature of an interaction that includes continuous variables [99] (**S12 Table**). In the Results section, we refer to “younger participants” and to “mature participants.” In each of these instances, the exact cutoff value expressed in age in months (as well as age in years in parenthesis) can be found in **S12 Table**. The table contains the lower threshold JNT value and the upper threshold JNT value, which are expressed in the units of the moderator (i.e., age in months and age in years). For the analyses described in the main text after controlling for gender, please see **S13 and S14 Tables**.

## Supporting information

S1 Table Gender and mean age (standard deviation in parentheses) during the first (Time 1, top half) and the second (Time 2, bottom half) assessment. (DOCX)

S2 Table Table depicting the results of the main text using a different neurotransmitter quantification method (MRS-Eq 2; see Materials and methods section). All values concern the interaction term between age and the neurotransmitter, as labeled in the first column. The models that included general intelligence as a covariate are labeled accordingly in the first column. df = degrees of freedom; P = *P* value; se = standard error; t = T-statistic; β = standardized regression coefficient.
(DOCX)

S3 Table Table depicting the results of the main text using a different neurotransmitter quantification method (MRS-Eq 2; see Materials and methods section) except that the dependent variable is the “numerical operations score”. All values concern the interaction term between age and the neurotransmitter, as labeled in the first column. The models that included general intelligence as a covariate are labeled accordingly in the first column. df = degrees of freedom; P = *P* value; se = standard error; t = T-statistic; β = standardized regression coefficient.
(DOCX)

S4 Table Table depicting the results of the main text using a different neurotransmitter quantification method (MRS-Eq 2; see Materials and methods section) except that the dependent variable is the “mathematical reasoning score”. All values concern the interaction term between age and the neurotransmitter, as labeled in the first column. The models that included general intelligence as a covariate are labeled accordingly in the first column. df = degrees of freedom; P = *P* value; se = standard error; t = T-statistic; β = standardized regression coefficient.
(DOCX)

S5 Table Table depicting the results of the main text using a different neurotransmitter quantification method (MRS-Eq 2; see Materials and methods section) except that the dependent variable is the “tempo score”. All values concern the interaction term between age and the neurotransmitter, as labeled in the first column. The models that included general intelligence as a covariate are labeled accordingly in the first column. df = degrees of freedom; P = *P* value; se = standard error; t = T-statistic; β = standardized regression coefficient.
(DOCX)

S6 Table S6–S8 Tables differ from corresponding S3–S5 Tables in respect to the neurotransmitter quantification method used, in that for S3–S5 Tables, we used the MRS-Eq 2, and for S6–S8 Tables, we used the MRS-Eq 1 (see Materials and methods section). Table depicting the results of the main text except that the dependent variable is the “numerical operations score”. All values concern the interaction term between age and the neurotransmitter, as labeled in the first column. The models that included general intelligence as a covariate are labeled accordingly in the first column. df = degrees of freedom; P = *P* value; se = standard error; t = T-statistic; β = standardized regression coefficient.
(DOCX)

S7 Table Table depicting the results of the main text except that the dependent variable is the “mathematical reasoning score”. All values concern the interaction term between age and the neurotransmitter, as labeled in the first column. The models that included general intelligence as a covariate are labeled accordingly in the first column. df = degrees of freedom; P = *P* value; se = standard error; t = T-statistic; β = standardized regression coefficient.
(DOCX)

S8 Table Table depicting the results of the main text except that the dependent variable is the “tempo score”. All values concern the interaction term between age and the neurotransmitter, as labeled in the first column. The models that included general intelligence as a covariate are labeled accordingly in the first column. df = degrees of freedom; P = *P* value; se = standard error; t = T-statistic; β = standardized regression coefficient.
(DOCX)

S9 Table Results from the Breusch–Pagan test assessing the presence of heteroscedasticity. P = *P* value; T = t-Statistic. The models correspond to the ones presented in the main text. For brevity, we label each column merely by the name of the neurotransmitter value. The models that included general intelligence as a covariate are labeled accordingly in the first column.
(DOCX)

S10 Table Table depicting the results of the assumption of residual normality using the Shapiro–Wilk test. P = *P* value; se = standard error; W = Shapiro–Wilk statistic. The first 2 value columns refer to the results when MRS-Eq 1 was used, and the last 2 value columns refer to the results when MRS-Eq 2 was used.
(DOCX)

S11 Table Table depicting the results of the multicollinearity assumption using the VIF. The values presented concern the maximum variance inflation factor from all the predictors. The first VIF column refers to the results when MRS-Eq 1 was used, and the second VIF column refers to the results when MRS-Eq 2 was used. In the prediction analyses (last 4 rows), we did not consider the VIF of age at Time 2, as age at Time 2 and age at Time 1 are expected to be very highly correlated. VIF = variance inflation factor.
(DOCX)

S12 Table Table depicting the boundaries of the JNT (expressed in age in months and age in years in parenthesis after rounding). When no values are shown, it means that no JNT value was obtained. JNT (L) = JNT lower threshold, a significant relationship existed between the neurotransmitter measure and MA for individuals with age below this threshold. JNT (U) = JNT upper threshold, a significant relationship existed between the neurotransmitter measure and MA for individuals with age above this threshold. JNT = Johnson–Neyman Technique; MA = mathematical achievement.
(DOCX)

S13 Table Table depicting the results of the main text using a different neurotransmitter quantification method (MRS-Eq 2; see Materials and methods section) when controlling for gender. All values concern the interaction term between age and the neurotransmitter, as labeled in the first column. df = degrees of freedom; P = *P* value; se = standard error; t = T-statistic; β = standardized regression coefficient.
(DOCX)

S14 Table Table depicting the results of the main text when controlling for gender. All values concern the interaction term between age and the neurotransmitter, as labeled in the first column. df = degrees of freedom; P = *P* value; se = standard error; t = T-statistic; β = standardized regression coefficient.
(DOCX)

S1 Fig The average spectrum from each of the 5 groups separately at Time 1. The spectrum thickness corresponds to ± 1 SD from the mean. (**A)** MFG 6-year-olds; (**B)** IPS 6-year-olds; (**C)** MFG 10-year-olds; (**D)** IPS 10-year-olds; (**E)** MFG 14-year-olds; (**F)** IPS 14-year-olds; (**G)** MFG 16-year-olds; (**H)** IPS 16-year-olds; (**I)** MFG 18+-year-olds; (**J)** IPS 18+ year-olds. IPS = intraparietal sulcus; MFG = middle frontal gyrus; SD = standard deviation.
(DOCX)

S2 Fig Positions of the 2 regions for the MRS displayed in a T1-weighted image for (**A**) IPS and (**B**) MFG are shown on axial and sagittal slices, respectively. IPS = intraparietal sulcus; MFG = middle frontal gyrus; MRS = magnetic resonance spectroscopy.
(DOCX)

S1 Text Additional information regarding the neurochemicals included in the basis set and how macromolecules were handled. The chemicals in the basis set that were automatically extracted from the analysis pipeline were as follows: GABA, glutamate, glutamine, alanine, ascorbate, aspartate, creatine, phosphocreatine, creatine+phosphocreatine, glucose, glycerophosphocholine, phosphocholine, glutathione, inositol, scyllo-Inositol, scyllo, lactate, phosphoethanolamine, NAA, NAAG, taurine, phophocholine+glycerophosphocholine, NAA+NAAG, glutamine+glutamate, and glucose+taurine. LCModel-simulated macromolecule resonances were included in the basis set: Macromolecule 09, Macromolecule 12, Macromolecule 14, Macromolecule 17, and Macromolecule 20.
(DOCX)

S1 Data An excel spreadsheet (S1_Data.xlsx) representing the data underling Fig 1. (XLSX)

S2 Data An excel spreadsheet (S2_Data.xlsx) representing the data underling Fig 2. (XLSX)

S3 Data An excel spreadsheet (S3_Data.xlsx) representing the data underling Fig 3. (XLSX)

## Abbreviations

CRLB: Cramér–Rao lower bound
CSF: cerebrospinal fluid
GABA: gamma-aminobutyric acid
GM: gray matter
IPS: intraparietal sulcus
JNT: Johnson–Neyman Technique
MA: mathematical achievement
MFG: middle frontal gyrus
MRS: magnetic resonance spectroscopy
se: standard error
SNR: signal-to-noise ratio
TE: echo time
TR: repetition time
VAPOR: variable power RF pulses with optimized relaxation delays
VeSPA: versatile simulation, pulses, and analysis
WASI II: Wechsler Abbreviated Scale of Intelligence
WIAT: Wechsler Individual Achievement Test
WM: white matter
