# Supplementary material for: Predicting learning and achievement using GABA and glutamate concentrations in human development
Source: PLoS Biol. 2021 Jul 22;19(7):e3001325. doi: 10.1371/journal.pbio.3001325 (PMC8297926; doi:10.1371/journal.pbio.3001325)
Supplement: S1 Table — (DOCX) [file pbio.3001325.s001.docx]

**S1 Table. Gender and mean age (standard deviation in parentheses) during the first (Time 1, top half) and the second (Time 2, bottom half) assessment.**

| **Group** | **Females** | **Males** | **Age (months)** |
| --- | --- | --- | --- |
| First Assessment (Time 1) | | | |
| 6 year-olds | 28 | 23 | 78.02 (3.51) |
| 10 year-olds | 27 | 24 | 125.35 (3.83) |
| 14 year-olds | 25 | 25 | 172.82 (3.96) |
| 16 year-olds | 26 | 23 | 202.51 (4.30) |
| 18+ year-olds | 21 | 33 | 226.65 (7.43) |
| Second Assessment (Time 2) | | | |
| 6 year-olds | 22 | 21 | 98.26 (4.39) |
| 10 year-olds | 22 | 18 | 147.53 (5.86) |
| 14 year-olds | 16 | 19 | 193.57 (4.82) |
| 16 year-olds | 12 | 13 | 221.72 (6.07) |
| 18+ year-olds | 12 | 22 | 247.15 (7.82) |
