# Supplementary material for: Predicting learning and achievement using GABA and glutamate concentrations in human development
Source: PLoS Biol. 2021 Jul 22;19(7):e3001325. doi: 10.1371/journal.pbio.3001325 (PMC8297926; doi:10.1371/journal.pbio.3001325)
Supplement: S2 Table — All values concern the interaction term between age and the neurotransmitter, as labeled in the first column. The models that included general intelligence as a covariate are labeled accordingly in the first column. df = degrees of freedom; P = P value; se = standard error; t = T-statistic; β = standardized regression coefficient. (DOCX) [file pbio.3001325.s002.docx]

**S2 Table. Table depicting the results of the main text using a different neurotransmitter quantification method (MRS-Eq 2; see Materials and methods section).** All values concern the interaction term between age and the neurotransmitter, as labeled in the first column. The models that included general intelligence as a covariate are labeled accordingly in the first column. df = degrees of freedom; P = *P* value; se = standard error; t = T-statistic; β = standardized regression coefficient.

| **First assessment (Time 1)** | | | | | |
| --- | --- | --- | --- | --- | --- |
|  | df | β | t | se | P |
| GLUIPS*age | 225 | 0.14 | 4.97 | 0.03 | <.0001 |
| GABAIPS*age | 224 | -0.11 | -4.30 | 0.03 | <.0001 |
| GLUMFG*age | 218 | 0.15 | 4.68 | 0.03 | <.0001 |
| GABAMFG*age | 213 | -0.02 | -0.70 | 0.03 | 0.4817 |
| GLUIPS*age + Intelligence | 220 | 0.12 | 4.62 | 0.02 | <.0001 |
| GABAIPS*age + Intelligence | 220 | -0.11 | -4.58 | 0.02 | <.0001 |
| GLUMFG*age + Intelligence | 214 | 0.11 | 3.40 | 0.03 | 0.0008 |
| GABAMFG*age + Intelligence | 209 | -0.02 | -0.92 | 0.03 | 0.3588 |
| **Second assessment (Time 2)** | | | | | |
|  | df | β | t | se | P |
| GLUIPS*age | 159 | 0.20 | 5.05 | 0.04 | <.0001 |
| GABAIPS*age | 159 | -0.12 | -2.88 | 0.04 | 0.0046 |
| GLUMFG*age | 152 | 0.19 | 4.64 | 0.04 | <.0001 |
| GABAMFG*age | 152 | -0.06 | -1.80 | 0.03 | 0.0736 |
| GLUIPS*age + Intelligence | 158 | 0.16 | 4.21 | 0.04 | <.0001 |
| GABAIPS*age + Intelligence | 158 | -0.08 | -1.90 | 0.04 | 0.0599 |
| **Predict MA at Time 2 using predictors from Time 1** | | | | | |
|  | df | β | t | se | P |
| GLUIPS*age | 150 | 0.16 | 3.97 | 0.04 | 0.0001 |
| GABAIPS*age | 149 | -0.12 | -3.45 | 0.04 | 0.0007 |
| GLUMFG*age | 146 | 0.18 | 3.84 | 0.05 | 0.0002 |
| GABAMFG*age | 141 | 0.02 | 0.55 | 0.04 | 0.5822 |
