# Supplementary material for: Predicting learning and achievement using GABA and glutamate concentrations in human development
Source: PLoS Biol. 2021 Jul 22;19(7):e3001325. doi: 10.1371/journal.pbio.3001325 (PMC8297926; doi:10.1371/journal.pbio.3001325)
Supplement: S3 Table — All values concern the interaction term between age and the neurotransmitter, as labeled in the first column. The models that included general intelligence as a covariate are labeled accordingly in the first column. df = degrees of freedom; P = P value; se = standard error; t = T-statistic; β = standardized regression coefficient. (DOCX) [file pbio.3001325.s003.docx]

**S3 Table. Table depicting the results of the main text using a different neurotransmitter quantification method (MRS-Eq 2; see Materials and methods section) except that the dependent variable is the “numerical operations score”.** All values concern the interaction term between age and the neurotransmitter, as labeled in the first column. The models that included general intelligence as a covariate are labeled accordingly in the first column. df = degrees of freedom; P = *P* value; se = standard error; t = T-statistic; β = standardized regression coefficient.

| **First assessment (Time 1)** | | | | | |
| --- | --- | --- | --- | --- | --- |
|  | df | β | t | se | P |
| GLUIPS*age | 223 | 0.15 | 5.85 | 0.03 | <.0001 |
| GABAIPS*age | 223 | -0.08 | -3.36 | 0.03 | 0.0009 |
| GLUMFG*age | 217 | 0.13 | 4.42 | 0.03 | <.0001 |
| GABAMFG*age | 213 | -0.03 | -1.21 | 0.02 | 0.2270 |
| GLUIPS*age + Intelligence | 220 | 0.11 | 4.20 | 0.03 | <.0001 |
| GABAIPS*age + Intelligence | 218 | -0.08 | -3.54 | 0.02 | 0.0005 |
| GLUMFG*age + Intelligence | 213 | 0.09 | 2.98 | 0.03 | 0.0032 |
| GABAMFG*age + Intelligence | 208 | -0.03 | -1.30 | 0.03 | 0.1948 |
| **Second assessment (Time 2)** | | | | | |
|  | df | β | t | se | P |
| GLUIPS*age | 159 | 0.18 | 5.13 | 0.04 | <.0001 |
| GABAIPS*age | 159 | -0.13 | -3.62 | 0.03 | 0.0004 |
| GLUMFG*age | 152 | 0.21 | 5.52 | 0.04 | <.0001 |
| GABAMFG*age | 152 | -0.08 | -2.36 | 0.03 | 0.0194 |
| GLUIPS*age + Intelligence | 158 | 0.15 | 4.34 | 0.03 | <.0001 |
| GABAIPS*age + Intelligence | 159 | -0.09 | -2.55 | 0.03 | 0.0117 |
| **Predict MA at Time 2 using predictors from Time 1** | | | | | |
|  | df | β | t | se | P |
| GLUIPS*age | 150 | 0.16 | 4.34 | 0.04 | <.0001 |
| GABAIPS*age | 149 | -0.14 | -3.72 | 0.04 | 0.0003 |
| GLUMFG*age | 146 | 0.18 | 3.76 | 0.05 | 0.0002 |
| GABAMFG*age | 141 | 0.00 | 0.09 | 0.03 | 0.9278 |
