# Supplementary material for: Predicting learning and achievement using GABA and glutamate concentrations in human development
Source: PLoS Biol. 2021 Jul 22;19(7):e3001325. doi: 10.1371/journal.pbio.3001325 (PMC8297926; doi:10.1371/journal.pbio.3001325)
Supplement: S4 Table — All values concern the interaction term between age and the neurotransmitter, as labeled in the first column. The models that included general intelligence as a covariate are labeled accordingly in the first column. df = degrees of freedom; P = P value; se = standard error; t = T-statistic; β = standardized regression coefficient. (DOCX) [file pbio.3001325.s004.docx]

**S4 Table. Table depicting the results of the main text using a different neurotransmitter quantification method (MRS-Eq 2; see Materials and methods section) except that the dependent variable is the “mathematical reasoning score”.** All values concern the interaction term between age and the neurotransmitter, as labeled in the first column. The models that included general intelligence as a covariate are labeled accordingly in the first column. df = degrees of freedom; P = *P* value; se = standard error; t = T-statistic; β = standardized regression coefficient.

| **First assessment (Time 1)** | | | | | |
| --- | --- | --- | --- | --- | --- |
|  | df | β | t | se | P |
| GLUIPS*age | 223 | 0.22 | 6.70 | 0.03 | <.0001 |
| GABAIPS*age | 222 | -0.20 | -5.93 | 0.03 | <.0001 |
| GLUMFG*age | 215 | 0.26 | 6.50 | 0.04 | <.0001 |
| GABAMFG*age | 213 | -0.07 | -2.03 | 0.04 | 0.0435 |
| GLUIPS*age + Intelligence | 220 | 0.16 | 5.27 | 0.03 | <.0001 |
| GABAIPS*age + Intelligence | 220 | -0.17 | -5.30 | 0.03 | <.0001 |
| GLUMFG*age + Intelligence | 212 | 0.17 | 3.76 | 0.04 | 0.0002 |
| GABAMFG*age + Intelligence | 209 | -0.08 | -2.26 | 0.04 | 0.0248 |
| **Second assessment (Time 2)** | | | | | |
|  | df | β | t | se | P |
| GLUIPS*age | 158 | 0.29 | 5.73 | 0.05 | <.0001 |
| GABAIPS*age | 157 | -0.23 | -4.05 | 0.06 | 0.0001 |
| GLUMFG*age | 150 | 0.27 | 5.33 | 0.05 | <.0001 |
| GABAMFG*age | 152 | -0.10 | -2.36 | 0.04 | 0.0194 |
| GLUIPS*age + Intelligence | 155 | 0.24 | 4.92 | 0.05 | <.0001 |
| GABAIPS*age + Intelligence | 156 | -0.19 | -3.23 | 0.06 | 0.0015 |
| **Predict MA at Time 2 using predictors from Time 1** | | | | | |
|  | df | β | t | se | P |
| GLUIPS*age | 149 | 0.27 | 5.74 | 0.05 | <.0001 |
| GABAIPS*age | 148 | -0.20 | -4.32 | 0.05 | <.0001 |
| GLUMFG*age | 146 | 0.27 | 4.63 | 0.06 | <.0001 |
| GABAMFG*age | 141 | 0.00 | -0.10 | 0.05 | 0.9166 |
