# Supplementary material for: Predicting learning and achievement using GABA and glutamate concentrations in human development
Source: PLoS Biol. 2021 Jul 22;19(7):e3001325. doi: 10.1371/journal.pbio.3001325 (PMC8297926; doi:10.1371/journal.pbio.3001325)
Supplement: S5 Table — All values concern the interaction term between age and the neurotransmitter, as labeled in the first column. The models that included general intelligence as a covariate are labeled accordingly in the first column. df = degrees of freedom; P = P value; se = standard error; t = T-statistic; β = standardized regression coefficient. (DOCX) [file pbio.3001325.s005.docx]

**S5 Table. Table depicting the results of the main text using a different neurotransmitter quantification method (MRS-Eq 2; see Materials and methods section) except that the dependent variable is the “tempo score”.** All values concern the interaction term between age and the neurotransmitter, as labeled in the first column. The models that included general intelligence as a covariate are labeled accordingly in the first column. df = degrees of freedom; P = *P* value; se = standard error; t = T-statistic; β = standardized regression coefficient.

| **First assessment (Time 1)** | | | | | |
| --- | --- | --- | --- | --- | --- |
|  | df | β | t | se | P |
| GLUIPS*age | 222 | 0.11 | 3.20 | 0.03 | 0.0016 |
| GABAIPS*age | 222 | -0.06 | -1.73 | 0.03 | 0.0851 |
| GLUMFG*age | 215 | 0.10 | 2.65 | 0.04 | 0.0086 |
| GABAMFG*age | 211 | -0.02 | -0.60 | 0.03 | 0.5462 |
| GLUIPS*age + Intelligence | 218 | 0.08 | 2.27 | 0.03 | 0.0243 |
| GABAIPS*age + Intelligence | 216 | -0.06 | -1.74 | 0.03 | 0.0830 |
| GLUMFG*age + Intelligence | 211 | 0.07 | 1.76 | 0.04 | 0.0793 |
| GABAMFG*age + Intelligence | 206 | -0.02 | -0.53 | 0.03 | 0.5993 |
| **Second assessment (Time 2)** | | | | | |
|  | df | β | t | se | P |
| GLUIPS*age | 157 | 0.13 | 2.89 | 0.04 | 0.0044 |
| GABAIPS*age | 158 | -0.05 | -1.23 | 0.04 | 0.2208 |
| GLUMFG*age | 151 | 0.07 | 1.51 | 0.04 | 0.1321 |
| GABAMFG*age | 151 | -0.04 | -1.02 | 0.04 | 0.3106 |
| GLUIPS*age + Intelligence | 156 | 0.10 | 2.26 | 0.04 | 0.0253 |
| GABAIPS*age + Intelligence | 158 | 0.00 | -0.08 | 0.05 | 0.9357 |
| **Predict MA at Time 2 using predictors from Time 1** | | | | | |
|  | df | β | t | se | P |
| GLUIPS*age | 149 | 0.07 | 1.69 | 0.04 | 0.0935 |
| GABAIPS*age | 148 | -0.07 | -1.77 | 0.04 | 0.0787 |
| GLUMFG*age | 145 | 0.07 | 1.39 | 0.05 | 0.1673 |
| GABAMFG*age | 140 | -0.02 | -0.38 | 0.04 | 0.7080 |
