# Supplementary material for: Predicting learning and achievement using GABA and glutamate concentrations in human development
Source: PLoS Biol. 2021 Jul 22;19(7):e3001325. doi: 10.1371/journal.pbio.3001325 (PMC8297926; doi:10.1371/journal.pbio.3001325)
Supplement: S6 Table — Table depicting the results of the main text except that the dependent variable is the “numerical operations score”. All values concern the interaction term between age and the neurotransmitter, as labeled in the first column. The models that included general intelligence as a covariate are labeled accordingly in the first column. df = degrees of freedom; P = P value; se = standard error; t = T-statistic; β = standardized regression coefficient. (DOCX) [file pbio.3001325.s006.docx]

**S6 Table.** **S6-S8 Tables differ from corresponding S3-S5 Tables in respect to the neurotransmitter quantification method used, in that for S3-S5 Tables, we used the MRS-Eq 2, and for S6-S8 Tables, we used the MRS-Eq 1 (see Materials and methods section).** Table depicting the results of the main text except that the dependent variable is the “numerical operations score”. All values concern the interaction term between age and the neurotransmitter, as labeled in the first column. The models that included general intelligence as a covariate are labeled accordingly in the first column. df = degrees of freedom; P = *P* value; se = standard error; t = T-statistic; β = standardized regression coefficient.

| **First assessment (Time 1)** | | | | | |
| --- | --- | --- | --- | --- | --- |
|  | df | β | t | se | P |
| GLUIPS*age | 223 | 0.14 | 5.45 | 0.03 | <.0001 |
| GABAIPS*age | 223 | -0.11 | -4.25 | 0.03 | <.0001 |
| GLUMFG*age | 220 | 0.09 | 3.07 | 0.03 | 0.0024 |
| GABAMFG*age | 215 | -0.03 | -1.21 | 0.03 | 0.2283 |
| GLUIPS*age + Intelligence | 220 | 0.11 | 3.98 | 0.03 | 0.0001 |
| GABAIPS*age + Intelligence | 218 | -0.10 | -4.08 | 0.02 | 0.0001 |
| GLUMFG*age + Intelligence | 215 | 0.06 | 2.02 | 0.03 | 0.0443 |
| GABAMFG*age + Intelligence | 210 | -0.04 | -1.48 | 0.03 | 0.1393 |
| **Second assessment (Time 2)** | | | | | |
|  | df | β | t | se | P |
| GLUIPS*age | 159 | 0.15 | 4.30 | 0.03 | <.0001 |
| GABAIPS*age | 159 | -0.16 | -4.81 | 0.03 | <.0001 |
| GLUMFG*age | 153 | 0.17 | 4.04 | 0.04 | 0.0001 |
| GABAMFG*age | 153 | -0.11 | -3.57 | 0.03 | 0.0005 |
| GLUIPS*age + Intelligence | 158 | 0.11 | 3.49 | 0.03 | 0.0006 |
| GABAIPS*age + Intelligence | 159 | -0.11 | -3.52 | 0.03 | 0.0006 |
| **Predict MA at Time 2 using predictors from Time 1** | | | | | |
|  | df | β | t | se | P |
| GLUIPS*age | 150 | 0.14 | 3.47 | 0.04 | 0.0007 |
| GABAIPS*age | 149 | -0.18 | -4.56 | 0.04 | <.0001 |
| GLUMFG*age | 147 | 0.13 | 2.70 | 0.05 | 0.0078 |
| GABAMFG*age | 144 | 0.00 | -0.01 | 0.04 | 0.9945 |
