# Supplementary material for: Predicting learning and achievement using GABA and glutamate concentrations in human development
Source: PLoS Biol. 2021 Jul 22;19(7):e3001325. doi: 10.1371/journal.pbio.3001325 (PMC8297926; doi:10.1371/journal.pbio.3001325)
Supplement: S7 Table — All values concern the interaction term between age and the neurotransmitter, as labeled in the first column. The models that included general intelligence as a covariate are labeled accordingly in the first column. df = degrees of freedom; P = P value; se = standard error; t = T-statistic; β = standardized regression coefficient. (DOCX) [file pbio.3001325.s007.docx]

**S7 Table. Table depicting the results of the main text except that the dependent variable is the “mathematical reasoning score”.** All values concern the interaction term between age and the neurotransmitter, as labeled in the first column. The models that included general intelligence as a covariate are labeled accordingly in the first column. df = degrees of freedom; P = *P* value; se = standard error; t = T-statistic; β = standardized regression coefficient.

| **First assessment (Time 1)** | | | | | |
| --- | --- | --- | --- | --- | --- |
|  | df | β | t | se | P |
| GLUIPS*age | 224 | 0.17 | 4.43 | 0.04 | <.0001 |
| GABAIPS*age | 222 | -0.23 | -7.20 | 0.03 | <.0001 |
| GLUMFG*age | 220 | 0.17 | 3.58 | 0.05 | 0.0004 |
| GABAMFG*age | 214 | -0.11 | -3.08 | 0.03 | 0.0023 |
| GLUIPS*age + Intelligence | 220 | 0.13 | 4.23 | 0.03 | <.0001 |
| GABAIPS*age + Intelligence | 220 | -0.19 | -6.29 | 0.03 | <.0001 |
| GLUMFG*age + Intelligence | 215 | 0.12 | 2.68 | 0.04 | 0.0080 |
| GABAMFG*age + Intelligence | 211 | -0.08 | -1.99 | 0.04 | 0.0474 |
| **Second assessment (Time 2)** | | | | | |
|  | df | β | t | se | P |
| GLUIPS*age | 158 | 0.27 | 5.35 | 0.05 | <.0001 |
| GABAIPS*age | 157 | -0.27 | -5.03 | 0.05 | <.0001 |
| GLUMFG*age | 151 | 0.23 | 4.15 | 0.06 | 0.0001 |
| GABAMFG*age | 152 | -0.13 | -3.61 | 0.04 | 0.0004 |
| GLUIPS*age + Intelligence | 155 | 0.22 | 4.51 | 0.05 | <.0001 |
| GABAIPS*age + Intelligence | 156 | -0.22 | -3.98 | 0.05 | 0.0001 |
| **Predict MA at Time 2 using predictors from Time 1** | | | | | |
|  | df | β | t | se | P |
| GLUIPS*age | 149 | 0.24 | 4.87 | 0.05 | <.0001 |
| GABAIPS*age | 148 | -0.23 | -5.28 | 0.04 | <.0001 |
| GLUMFG*age | 146 | 0.20 | 3.64 | 0.06 | 0.0004 |
| GABAMFG*age | 143 | 0.01 | 0.21 | 0.06 | 0.8368 |
