# Supplementary material for: Predicting learning and achievement using GABA and glutamate concentrations in human development
Source: PLoS Biol. 2021 Jul 22;19(7):e3001325. doi: 10.1371/journal.pbio.3001325 (PMC8297926; doi:10.1371/journal.pbio.3001325)
Supplement: S8 Table — All values concern the interaction term between age and the neurotransmitter, as labeled in the first column. The models that included general intelligence as a covariate are labeled accordingly in the first column. df = degrees of freedom; P = P value; se = standard error; t = T-statistic; β = standardized regression coefficient. (DOCX) [file pbio.3001325.s008.docx]

**S8 Table. Table depicting the results of the main text except that the dependent variable is the “tempo score”.** All values concern the interaction term between age and the neurotransmitter, as labeled in the first column. The models that included general intelligence as a covariate are labeled accordingly in the first column. df = degrees of freedom; P = *P* value; se = standard error; t = T-statistic; β = standardized regression coefficient.

| **First assessment (Time 1)** | | | | | |
| --- | --- | --- | --- | --- | --- |
|  | df | β | t | se | P |
| GLUIPS*age | 222 | 0.12 | 3.46 | 0.03 | 0.0006 |
| GABAIPS*age | 222 | -0.07 | -2.01 | 0.03 | 0.0460 |
| GLUMFG*age | 217 | 0.08 | 2.32 | 0.04 | 0.0213 |
| GABAMFG*age | 213 | -0.01 | -0.46 | 0.03 | 0.6437 |
| GLUIPS*age + Intelligence | 218 | 0.09 | 2.66 | 0.03 | 0.0084 |
| GABAIPS*age + Intelligence | 216 | -0.06 | -1.83 | 0.03 | 0.0682 |
| GLUMFG*age + Intelligence | 213 | 0.06 | 1.50 | 0.04 | 0.1341 |
| GABAMFG*age + Intelligence | 208 | -0.01 | -0.46 | 0.03 | 0.6491 |
| **Second assessment (Time 2)** | | | | | |
|  | df | β | t | se | P |
| GLUIPS*age | 157 | 0.11 | 2.58 | 0.04 | 0.0106 |
| GABAIPS*age | 158 | -0.07 | -1.60 | 0.04 | 0.1126 |
| GLUMFG*age | 152 | 0.05 | 1.12 | 0.05 | 0.2637 |
| GABAMFG*age | 152 | -0.03 | -0.82 | 0.04 | 0.4129 |
| GLUIPS*age + Intelligence | 156 | 0.08 | 1.98 | 0.04 | 0.0491 |
| GABAIPS*age + Intelligence | 158 | -0.02 | -0.33 | 0.05 | 0.7381 |
| **Predict MA at Time 2 using predictors from Time 1** | | | | | |
|  | df | β | t | se | P |
| GLUIPS*age | 149 | 0.07 | 1.80 | 0.04 | 0.0735 |
| GABAIPS*age | 148 | -0.08 | -2.07 | 0.04 | 0.0401 |
| GLUMFG*age | 146 | 0.07 | 1.37 | 0.05 | 0.1743 |
| GABAMFG*age | 142 | 0.00 | -0.05 | 0.04 | 0.9577 |
