# Supplementary material for: Predicting learning and achievement using GABA and glutamate concentrations in human development
Source: PLoS Biol. 2021 Jul 22;19(7):e3001325. doi: 10.1371/journal.pbio.3001325 (PMC8297926; doi:10.1371/journal.pbio.3001325)
Supplement: S9 Table — The models correspond to the ones presented in the main text. For brevity, we label each column merely by the name of the neurotransmitter value. The models that included general intelligence as a covariate are labeled accordingly in the first column. (DOCX) [file pbio.3001325.s009.docx]

**S9 Table. Results from the Breusch-Pagan test assessing the presence of heteroscedasticity. P = *P* value; T = t-Statistic.** The models correspond to the ones presented in the main text. For brevity, we label each column merely by the name of the neurotransmitter value. The models that included general intelligence as a covariate are labeled accordingly in the first column.

| **First assessment (Time 1)** | | |
| --- | --- | --- |
|  | T | P |
| GLUIPS*age | 19.18 | 0.00025 |
| GABAIPS*age | 14.31 | 0.00251 |
| GLUMFG*age | 18.92 | 0.00028 |
| GABAMFG*age | 18.89 | 0.00029 |
| GLUIPS*age + Intelligence | 15.37 | 0.00399 |
| GABAIPS*age + Intelligence | 12.49 | 0.01408 |
| GLUMFG*age + Intelligence | 14.31 | 0.00638 |
| GABAMFG*age + Intelligence | 14.41 | 0.00610 |
| **Second assessment (Time 2)** | | |
|  | T | P |
| GLUIPS*age | 10.03 | 0.01833 |
| GABAIPS*age | 9.19 | 0.02691 |
| GLUMFG*age | 16.63 | 0.00084 |
| GABAMFG*age | 8.04 | 0.04511 |
| GLUIPS*age + Intelligence | 9.65 | 0.04673 |
| GABAIPS*age + Intelligence | 9.91 | 0.04190 |
| **Predict MA at Time 2 using predictors from Time 1** | | |
|  | T | P |
| GLUIPS*age | 14.23 | 0.00660 |
| GABAIPS*age | 15.34 | 0.00404 |
| GLUMFG*age | 13.36 | 0.00963 |
| GABAMFG*age | 11.02 | 0.02639 |
