# Supplementary material for: Predicting learning and achievement using GABA and glutamate concentrations in human development
Source: PLoS Biol. 2021 Jul 22;19(7):e3001325. doi: 10.1371/journal.pbio.3001325 (PMC8297926; doi:10.1371/journal.pbio.3001325)
Supplement: S10 Table — P = P value; se = standard error; W = Shapiro–Wilk statistic. The first 2 value columns refer to the results when MRS-Eq 1 was used, and the last 2 value columns refer to the results when MRS-Eq 2 was used. (DOCX) [file pbio.3001325.s010.docx]

**S10 Table. Table depicting the results of the assumption of residual normality using the Shapiro-Wilk test.** P = *P* value; se = standard error; W = Shapiro-Wilk statistic. The first 2 value columns refer to the results when MRS-Eq 1 was used, and the last 2 value columns refer to the results when MRS-Eq 2 was used.

| **First assessment (Time 1)** | | | | |
| --- | --- | --- | --- | --- |
|  | W | P | W | P |
| GLUIPS*age | 0.99 | 0.53 | 0.99 | 0.61 |
| GABAIPS*age | 0.99 | 0.54 | 0.99 | 0.51 |
| GLUMFG*age | 0.99 | 0.63 | 1.00 | 0.85 |
| GABAMFG*age | 0.99 | 0.12 | 0.99 | 0.12 |
| GLUIPS*age + Intelligence | 0.99 | 0.28 | 0.99 | 0.54 |
| GABAIPS*age + Intelligence | 0.99 | 0.27 | 0.99 | 0.14 |
| GLUMFG*age + Intelligence | 0.99 | 0.19 | 0.99 | 0.26 |
| GABAMFG*age + Intelligence | 0.99 | 0.02 | 0.98 | 0.02 |
| **Second assessment (Time 2)** | | | | |
|  | W | P | W | P |
| GLUIPS*age | 0.99 | 0.85 | 0.99 | 0.80 |
| GABAIPS*age | 1.00 | 0.97 | 1.00 | 0.95 |
| GLUMFG*age | 1.00 | 0.98 | 1.00 | 0.94 |
| GABAMFG*age | 1.00 | 0.95 | 1.00 | 0.91 |
| GLUIPS*age + Intelligence | 0.99 | 0.43 | 0.99 | 0.48 |
| GABAIPS*age + Intelligence | 0.99 | 0.77 | 0.99 | 0.72 |
| **Predict MA at Time 2 using predictors from Time 1** | | | | |
|  | W | P | W | P |
| GLUIPS*age | 1.00 | 0.99 | 1.00 | 0.90 |
| GABAIPS*age | 0.99 | 0.65 | 0.99 | 0.75 |
| GLUMFG*age | 1.00 | 0.97 | 1.00 | 0.91 |
| GABAMFG*age | 1.00 | 0.95 | 1.00 | 0.95 |
