# Supplementary material for: Predicting learning and achievement using GABA and glutamate concentrations in human development
Source: PLoS Biol. 2021 Jul 22;19(7):e3001325. doi: 10.1371/journal.pbio.3001325 (PMC8297926; doi:10.1371/journal.pbio.3001325)
Supplement: S11 Table — The values presented concern the maximum variance inflation factor from all the predictors. The first VIF column refers to the results when MRS-Eq 1 was used, and the second VIF column refers to the results when MRS-Eq 2 was used. In the prediction analyses (last 4 rows), we did not consider the VIF of age at Time 2, as age at Time 2 and age at Time 1 are expected to be very highly correlated. VIF = variance inflation factor. (DOCX) [file pbio.3001325.s011.docx]

**S11 Table. Table depicting the results of the multicollinearity assumption using the VIF.** The values presented concern the maximum variance inflation factor from all the predictors. The first VIF column refers to the results when MRS-Eq 1 was used, and the second VIF column refers to the results when MRS-Eq 2 was used. In the prediction analyses (last four rows), we did not consider the VIF of age at Time 2, as age at Time 2 and age at Time 1 are expected to be very highly correlated. VIF = variance inflation factor.

| **First assessment (Time 1)** | | |
| --- | --- | --- |
|  | VIF | VIF |
| GLUIPS*age | 1.48 | 1.74 |
| GABAIPS*age | 1.35 | 1.21 |
| GLUMFG*age | 1.43 | 1.67 |
| GABAMFG*age | 1.24 | 1.19 |
| GLUIPS*age + Intelligence | 3.00 | 3.14 |
| GABAIPS*age + Intelligence | 2.94 | 2.86 |
| GLUMFG*age + Intelligence | 3.00 | 3.19 |
| GABAMFG*age + Intelligence | 3.18 | 3.05 |
| **Second assessment (Time 2)** | | |
|  | VIF | VIF |
| GLUIPS*age | 1.55 | 1.80 |
| GABAIPS*age | 1.25 | 1.15 |
| GLUMFG*age | 1.34 | 1.49 |
| GABAMFG*age | 1.18 | 1.11 |
| GLUIPS*age + Intelligence | 2.64 | 2.85 |
| GABAIPS*age + Intelligence | 2.35 | 2.27 |
| **Predict MA at Time 2 using predictors from Time 1** | | |
|  | VIF | VIF |
| GLUIPS*age | 1.42 | 1.69 |
| GABAIPS*age | 1.31 | 1.18 |
| GLUMFG*age | 1.33 | 1.57 |
| GABAMFG*age | 1.15 | 1.12 |
