# Supplementary material for: Predicting learning and achievement using GABA and glutamate concentrations in human development
Source: PLoS Biol. 2021 Jul 22;19(7):e3001325. doi: 10.1371/journal.pbio.3001325 (PMC8297926; doi:10.1371/journal.pbio.3001325)
Supplement: S12 Table — When no values are shown, it means that no JNT value was obtained. JNT (L) = JNT lower threshold, a significant relationship existed between the neurotransmitter measure and MA for individuals with age below this threshold. JNT (U) = JNT upper threshold, a significant relationship existed between the neurotransmitter measure and MA for individuals with age above this threshold. JNT = Johnson–Neyman Technique; MA = mathematical achievement. (DOCX) [file pbio.3001325.s012.docx]

**S12 Table. Table depicting the boundaries of the JNT (expressed in age in months and age in years in parenthesis after rounding).** When no values are shown, it means that no JNT value was obtained. JNT (L) = JNT lower threshold, a significant relationship existed between the neurotransmitter measure and MA for individuals with age below this threshold. JNT (U) = JNT upper threshold, a significant relationship existed between the neurotransmitter measure and MA for individuals with age above this threshold. JNT = Johnson-Neyman Technique; MA = mathematical achievement.

|  | **First assessment (Time 1)** | |
| --- | --- | --- |
|  | **JNT (L)** | **JNT (U)** |
| **GLUIPS** | 159 (13) | 212 (18) |
| **GABAIPS** | 158 (13) | 204 (17) |
| **GLUMFG** | 127 (11) | 192 (16) |
| **GABAMFG** | - | - |
|  | **Second assessment (Time 2)** | |
|  | **JNT (L)** | **JNT (U)** |
| **GLUIPS** | 153 (13) | 210 (18) |
| **GABAIPS** | 180 (15) | 250 (21) |
| **GLUMFG** | 170 (14) | 234 (20) |
| **GABAMFG** | 150 (13) | - |
| **Predict MA at Time 2 using predictors from Time 1** | | |
|  | **JNT (L)** | **JNT (U)** |
| **GLUIPS** | 147 (12) | 230 (19) |
| **GABAIPS** | 154 (13) | 220 (18) |
| **GLUMFG** | 125 (10) | 215 (18) |
| **GABAMFG** | - | - |
