# Supplementary material for: Predicting learning and achievement using GABA and glutamate concentrations in human development
Source: PLoS Biol. 2021 Jul 22;19(7):e3001325. doi: 10.1371/journal.pbio.3001325 (PMC8297926; doi:10.1371/journal.pbio.3001325)
Supplement: S13 Table — All values concern the interaction term between age and the neurotransmitter, as labeled in the first column. df = degrees of freedom; P = P value; se = standard error; t = T-statistic; β = standardized regression coefficient. (DOCX) [file pbio.3001325.s013.docx]

**S13 Table. Table depicting the results of the main text using a different neurotransmitter quantification method (MRS-Eq 2; see Materials and methods section) when controlling for gender.** All values concern the interaction term between age and the neurotransmitter, as labeled in the first column. df = degrees of freedom; P = *P* value; se = standard error; t = T-statistic; β = standardized regression coefficient.

| **First assessment (Time 1)** | | | | | |
| --- | --- | --- | --- | --- | --- |
|  | df | β | t | se | P |
| GLUIPS*age + gender | 224 | 0.14 | 5.09 | 0.03 | <.0001 |
| GABAIPS*age + gender | 223 | -0.11 | -4.30 | 0.03 | <.0001 |
| GLUMFG*age + gender | 217 | 0.15 | 4.76 | 0.03 | <.0001 |
| GABAMFG*age + gender | 212 | -0.02 | -0.94 | 0.03 | 0.3508 |
| **Second assessment (Time 2)** | | | | | |
|  | df | β | t | se | P |
| GLUIPS*age + gender | 157 | 0.21 | 5.24 | 0.04 | <.0001 |
| GABAIPS*age + gender | 158 | -0.12 | -2.88 | 0.04 | 0.0045 |
| GLUMFG*age + gender | 151 | 0.19 | 4.66 | 0.04 | <.0001 |
| GABAMFG*age + gender | 151 | -0.07 | -2.04 | 0.03 | 0.0429 |
| **Predict MA at Time 2 using predictors from Time 1** | | | | | |
|  | df | β | t | se | P |
| GLUIPS*age + gender | 148 | 0.17 | 4.35 | 0.04 | <.0001 |
| GABAIPS*age + gender | 146 | -0.15 | -4.30 | 0.03 | <.0001 |
| GLUMFG*age + gender | 144 | 0.19 | 4.16 | 0.05 | 0.0001 |
| GABAMFG*age + gender | 140 | 0.02 | 0.42 | 0.04 | 0.6717 |
