# Supplementary material for: Predicting learning and achievement using GABA and glutamate concentrations in human development
Source: PLoS Biol. 2021 Jul 22;19(7):e3001325. doi: 10.1371/journal.pbio.3001325 (PMC8297926; doi:10.1371/journal.pbio.3001325)
Supplement: S14 Table — All values concern the interaction term between age and the neurotransmitter, as labeled in the first column. df = degrees of freedom; P = P value; se = standard error; t = T-statistic; β = standardized regression coefficient. (DOCX) [file pbio.3001325.s014.docx]

**S14 Table. Table depicting the results of the main text when controlling for gender.** All values concern the interaction term between age and the neurotransmitter, as labeled in the first column. df = degrees of freedom; P = *P* value; se = standard error; t = T-statistic; β = standardized regression coefficient.

| **First assessment (Time 1)** | | | | | |
| --- | --- | --- | --- | --- | --- |
|  | df | β | t | se | P |
| GLUIPS*age + gender | 224 | 0.13 | 4.47 | 0.03 | <.0001 |
| GABAIPS*age + gender | 223 | -0.14 | -5.39 | 0.03 | <.0001 |
| GLUMFG*age + gender | 219 | 0.11 | 3.61 | 0.03 | 0.0004 |
| GABAMFG*age + gender | 214 | -0.02 | -0.66 | 0.03 | 0.5119 |
| **Second assessment (Time 2)** | | | | | |
|  | df | β | t | se | P |
| GLUIPS*age + gender | 158 | 0.17 | 4.43 | 0.04 | <.0001 |
| GABAIPS*age + gender | 158 | -0.15 | -3.89 | 0.04 | 0.0001 |
| GLUMFG*age + gender | 152 | 0.16 | 3.58 | 0.04 | 0.0005 |
| GABAMFG*age + gender | 152 | -0.09 | -2.82 | 0.03 | 0.0055 |
| **Predict MA at Time 2 using predictors from Time 1** | | | | | |
|  | df | β | t | se | P |
| GLUIPS*age + gender | 148 | 0.14 | 3.80 | 0.04 | 0.0002 |
| GABAIPS*age + gender | 146 | -0.17 | -5.10 | 0.03 | <.0001 |
| GLUMFG*age + gender | 146 | 0.14 | 3.31 | 0.04 | 0.0012 |
| GABAMFG*age + gender | 142 | 0.02 | 0.49 | 0.04 | 0.6262 |
