# Supplementary material for: Predicting learning and achievement using GABA and glutamate concentrations in human development
Source: PLoS Biol. 2021 Jul 22;19(7):e3001325. doi: 10.1371/journal.pbio.3001325 (PMC8297926; doi:10.1371/journal.pbio.3001325)

**S1 Fig. The average spectrum from each of the five groups separately at Time 1.** The spectrum thickness corresponds to ± 1 standard deviation from the mean. (**A**) MFG 6 year-olds; (**B**) IPS 6 year-olds; (**C**) MFG 10 year-olds; (**D**) IPS 10 year-olds; (**E**) MFG 14 year-olds; (**F**) IPS 14 year-olds; (**G**) MFG 16 year-olds; (**H**) IPS 16 year-olds; (**I**) MFG 18+ year-olds; (**J**) IPS 18+ year-olds. IPS = intraparietal sulcus; MFG = middle frontal gyrus; SD = standard deviation.


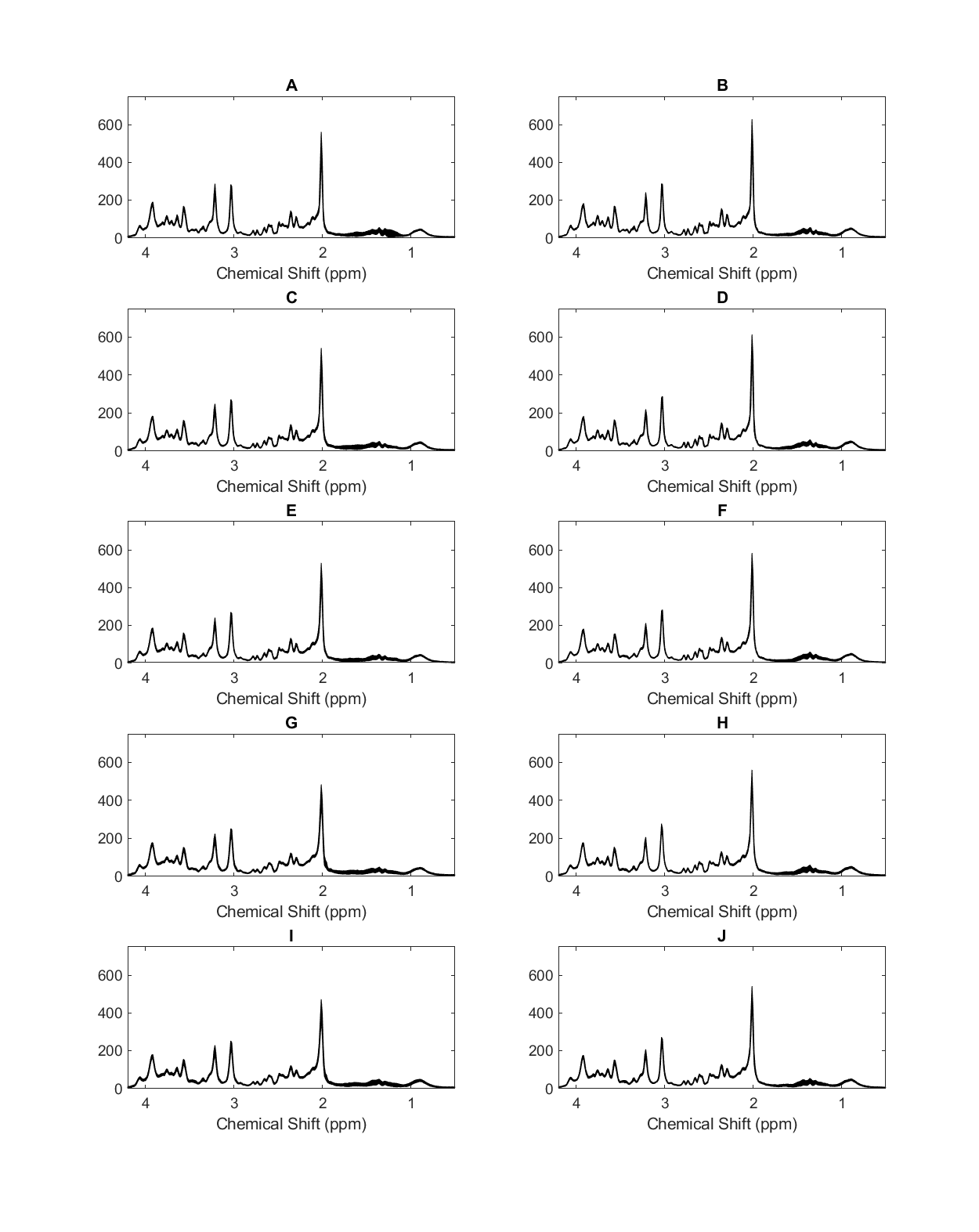

Supplement: S1 Fig — The spectrum thickness corresponds to ± 1 SD from the mean. (A) MFG 6-year-olds; (B) IPS 6-year-olds; (C) MFG 10-year-olds; (D) IPS 10-year-olds; (E) MFG 14-year-olds; (F) IPS 14-year-olds; (G) MFG 16-year-olds; (H) IPS 16-year-olds; (I) MFG 18+-year-olds; (J) IPS 18+ year-olds. IPS = intraparietal sulcus; MFG = middle frontal gyrus; SD = standard deviation. (DOCX) [file pbio.3001325.s015.docx]
