# Supplementary material for: Predicting learning and achievement using GABA and glutamate concentrations in human development
Source: PLoS Biol. 2021 Jul 22;19(7):e3001325. doi: 10.1371/journal.pbio.3001325 (PMC8297926; doi:10.1371/journal.pbio.3001325)

**S2 Fig.** Positions of the 2 regions for the MRS displayed in a T1-weighted image for (**A**) IPS, (**B**) MFG are shown on axial and sagittal slices, respectively. IPS = intraparietal sulcus; MFG = middle frontal gyrus; MRS = magnetic resonance spectroscopy.


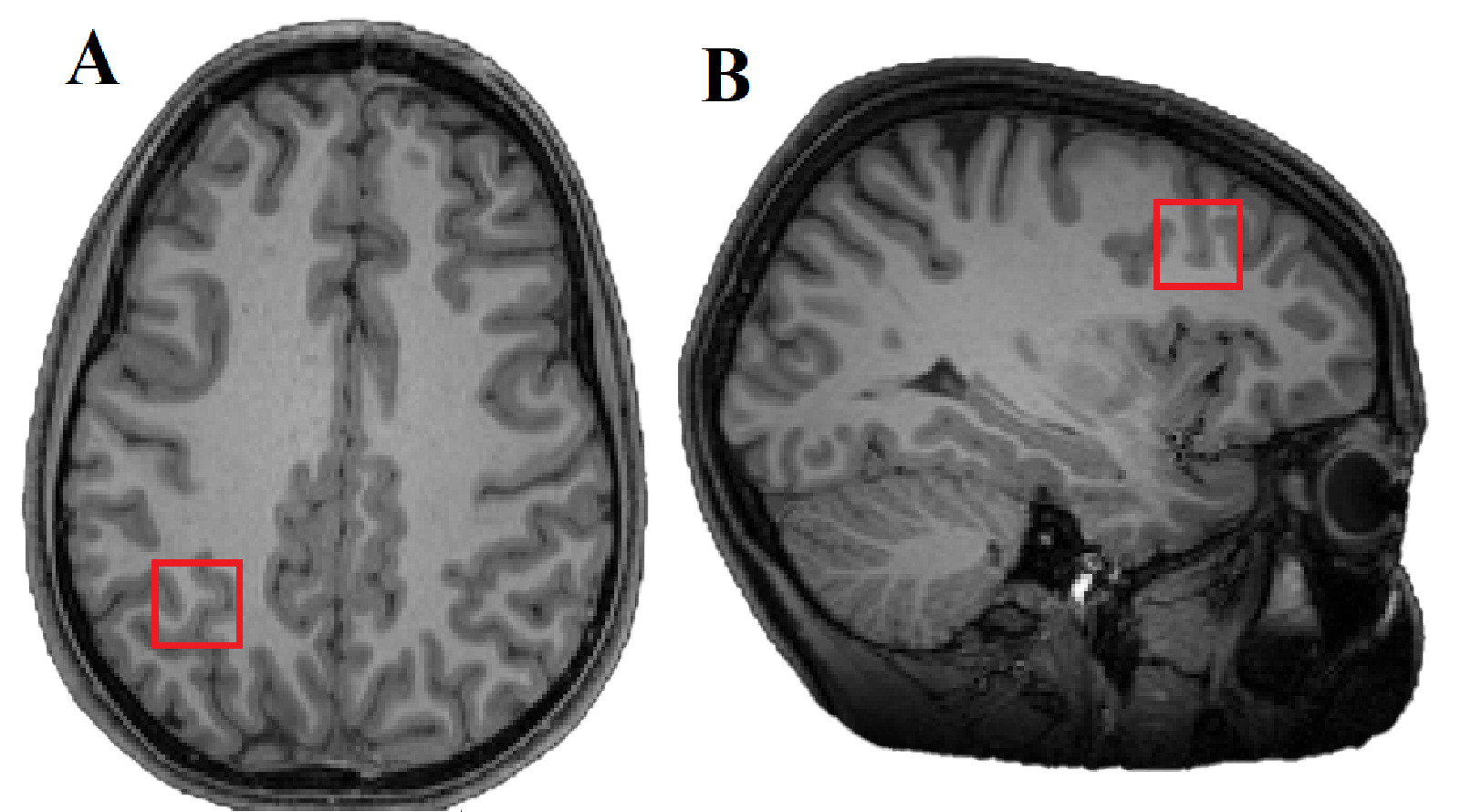

Supplement: S2 Fig — Positions of the 2 regions for the MRS displayed in a T1-weighted image for (A) IPS and (B) MFG are shown on axial and sagittal slices, respectively. IPS = intraparietal sulcus; MFG = middle frontal gyrus; MRS = magnetic resonance spectroscopy. (DOCX) [file pbio.3001325.s016.docx]
