# Supplementary material for: Predicting learning and achievement using GABA and glutamate concentrations in human development
Source: PLoS Biol. 2021 Jul 22;19(7):e3001325. doi: 10.1371/journal.pbio.3001325 (PMC8297926; doi:10.1371/journal.pbio.3001325)
Supplement: S1 Text — The chemicals in the basis set that were automatically extracted from the analysis pipeline were as follows: GABA, glutamate, glutamine, alanine, ascorbate, aspartate, creatine, phosphocreatine, creatine+phosphocreatine, glucose, glycerophosphocholine, phosphocholine, glutathione, inositol, scyllo-Inositol, scyllo, lactate, phosphoethanolamine, NAA, NAAG, taurine, phophocholine+glycerophosphocholine, NAA+NAAG, glutamine+glutamate, and glucose+taurine. LCModel-simulated macromolecule resonances were included in the basis set: Macromolecule 09, Macromolecule 12, Macromolecule 14, Macromolecule 17, and Macromolecule 20. (DOCX) [file pbio.3001325.s017.docx]

**S1 Text. Additional information regarding the neurochemicals included in the basis set and how macromolecules were handled.** The chemicals in the basis set that were automatically extracted from the analysis pipeline were as follows: GABA, glutamate, glutamine, alanine, ascorbate, aspartate, creatine, phosphocreatine, creatine+phosphocreatine, glucose, glycerophosphocholine, phosphocholine, glutathione, inositol, scyllo-Inositol, scyllo, lactate, phosphoethanolamine, NAA, NAAG, taurine, phophocholine+glycerophosphocholine, NAA+NAAG, glutamine+glutamate, glucose+taurine. LCModel‐simulated macromolecule resonances were included in the basis set: Macromolecule 09, Macromolecule 12, Macromolecule 14, Macromolecule 17, Macromolecule 20.
